# Ectopic Expression of the Rice Grain-Size-Affecting Gene GS5 in Maize Affects Kernel Size by Regulating Endosperm Starch Synthesis

**DOI:** 10.3390/genes13091542

**Published:** 2022-08-26

**Authors:** Guoqing Dong, Hanxian Xiong, Wanyong Zeng, Jinhua Li, Dengxiang Du

**Affiliations:** 1 School of Life Science and Technology, Wuhan Polytechnic University, Wuhan 430023, China; 2 National Key Laboratory of Crop Genetic Improvement, Huazhong Agricultural University, Wuhan 430070, China

**Keywords:** maize, transgenic, overexpression, *GS5*, seed starch

## Abstract

Maize is one of the most important food crops, and maize kernel is one of the important components of maize yield. Studies have shown that the rice *grain-size affecting gene GS5* increases the thousand-kernel weight by positively regulating the rice grain width and grain grouting rate. In this study, based on the *GS5* transgenic maize obtained through transgenic technology with specific expression in the endosperm, molecular assays were performed on the transformed plants. Southern blotting results showed that the *GS5* gene was integrated into the maize genome in a low copy number, and RT-PCR analysis showed that the exogenous *GS5* gene was normally and highly expressed in maize. The agronomic traits of two successive generations showed that certain lines were significantly improved in yield-related traits, and the most significant changes were observed in the OE-34 line, where the kernel width increased significantly by 8.99% and 10.96%, the 100-kernel weight increased by 14.10% and 10.82%, and the ear weight increased by 13.96% and 15.71%, respectively; however, no significant differences were observed in the plant height, ear height, kernel length, kernel row number, or kernel number. In addition, the overexpression of the *GS5* gene increased the grain grouting rate and affected starch synthesis in the rice grains. The kernels’ starch content in OE-25, OE-34, and OE-57 increased by 10.30%, 7.39%, and 6.39%, respectively. Scanning electron microscopy was performed to observe changes in the starch granule size, and the starch granule diameter of the transgenic line(s) was significantly reduced. RT-PCR was performed to detect the expression levels of related genes in starch synthesis, and the expression of these genes was generally upregulated. It was speculated that the exogenous *GS5* gene changed the size of the starch granules by regulating the expression of related genes in the starch synthesis pathway, thus increasing the starch content. The *trans-GS5* gene was able to be stably expressed in the hybrids with the genetic backgrounds of the four materials, with significant increases in the kernel width, 100-kernel weight, and ear weight. In this study, the maize kernel size was significantly increased through the endosperm-specific expression of the rice *GS5* gene, and good material for the functional analysis of the *GS5* gene was created, which was of great importance in theory and application.

## 1. Introduction

Maize is an important food crop, source of feed, and industrial raw material. With the expansion of the human habitat and the destruction of the natural environment, the amount of arable land has been irreversibly reduced. Increasing the yield per unit area of maize to ensure food security with the limited arable land is obviously an important initiative [1,2]. Maize yield is a complex quantitative trait controlled by various genes and environmental factors [3,4,5], among which photosynthesis, nitrogen assimilation, carbon allocation, plant stature, and other physiological processes are the basis of yield formation [6,7,8,9]. Maize kernels are the main harvest target, and the kernel yield comprises the number of plants per unit area, the number of kernels, and the 100-kernel weight, which is determined by and highly positively correlated with kernel length, kernel width, and kernel thickness [10,11,12].

Transgenic breeding refers to the use of genetic engineering methods to introduce single or multiple target genes into the recipient maize genome, where the target genes are stably expressed and bring new agronomic traits such as disease resistance, insect resistance, high yield, and high quality to maize [13,14,15]. This method is used in combination with conventional breeding methods and field evaluation methods to breed new maize varieties. The transgenic breeding of maize can break the reproductive barriers between different species, aggregate different favorable genes (e.g., disease resistance and high yield-related genes), and create targeted genetic traits in maize to significantly reduce the breeding cycle of new maize varieties and enhance the efficiency of maize genetic improvement [16,17,18]. In the last decade, many endogenous maize genes have exhibited overexpression in maize due to genetic engineering, and these maize lines have significantly improved yield. For example, AGPase is a key enzyme controlling starch synthesis in seeds [19], and the overexpression of the *shrunken-2* and *brittle-2* genes in maize significantly increased the AGPase activity, increased the starch content from 65% to 74%, and increased the 100-kernel weight by 15% [20,21]. Di et al. cloned the maize arginase *ZmArg* gene and introduced it into the inbred maize line K10, which significantly increased the yield per plant and 100-kernel weight by improving nitrogen storage efficiency in the maize [22]. Xie et al. overexpressed the *Zmdar1* and *Zmda1* genes in maize homozygous lines and obtained an increased ear number, significantly increased 100-kernel weight, and an increased plot yield by 15–22% [23]. The enhanced expression of the transcription factor *zmm28* of the MADS-box gene of maize resulted in improved plant growth, photosynthetic capacity, and nitrogen utilization in maize, which increased maize yield by 10% in different locations and years [24,25]. It is foreseeable that promoting research on the application, commercialization, and cultivation of different types of transgenic maize would be beneficial in reducing production costs, increasing yields, and improving economic efficiency [26,27,28,29].

Rice is one of the three major food crops and a model crop for the study of the Poaceae family [30,31]. Grain shape is one of the important factors determining the thousand-grain weight of rice [32]. To date, many grain-weight-related genes have been cloned, such as *GS3* [33], *TGW6* [34], and *GS9* [32], which control grain length and thousand-grain weight; *GW5* [35] and *GS5* [36], which control grain width and thousand-grain weight; and *GIF1* [37] and *WTG1* [38], which control seed grouting rate. It is of great significance to clone yield-related genes of rice, analyze their functions, and apply these genes to other monocotyledonous crops in the Poaceae family using genetic engineering approaches.

The *GS5* gene, located on chromosome No. 5 in rice, encodes serine carboxypeptidase-like proteins (SCPLs), and is a major gene that positively regulates rice seed width, thousand-grain weight, and grouting rate [36,39]. The overexpression of *GS5* increased rice seed length and yield, as well as promoting the expression of the *CDKA1*, *CAK1*, *CAK1A*, *CYCT1*, and *H1* genes in the mitotic G1/S transition of the cell cycle, which promoted glume cell division and increased the glume number [40]. Li et al. noted that the yield of other crops may, likewise, be improved if they contained the *GS5* gene. The introduction of the rice *GS5* gene into wheat led to a significant increase in grain width and thousand-grain weight in the transgenic line, with no effect on the other yield factors.

It is of utmost importance to breed new high-yielding maize varieties through genetic engineering. At present, a number of new insect- and herbicide-resistant transgenic maize varieties have been released [41,42,43]. However, our understanding of the regulatory network is limited, so there have been few reports on high-yielding transgenic maize. The rice *GS5* gene positively regulates grain width, grain weight, and grouting rate in rice and may improve yields if introduced into other crops.

## 2. Materials and Methods

### 2.1. Preparation of Transgenic Line(s)

According to the *Agrobacterium*-mediated gene transfer reported by Du et al., the vector *pHZM1N-PZmMRP-1::GS5* was transfected into the embryonic callus of the inbred maize line A188 [44]. The transfected calluses were selected under a blue light, and those with green-fluorescence expression were selected as transgenic-positive calluses. Calluses with green-fluorescence expression were obtained after three rounds of green-fluorescence screening and were then subjected to heat shock at 42 °C. Heat shock was performed three times for 2 h each. After 7 days in recovery culture, the calluses with eliminated green fluorescence were selected and transferred to the differentiation culture medium for differentiation. The regenerated plants were transferred to the rooting medium when they reached 3 cm. The marker-gene knockout was screened using phenotypic tests based on whether the regenerated plants grew cluster buds or whether they rooted, followed by a PCR assay using the primers GSR/5 and GS5F. The differentiated and regenerated plants were transferred to the rooting medium, hardened off, and transplanted in a greenhouse after sound root growth. Transgenic-positive plants were obtained using PCR analysis of the target genes. The T_0_ generation was harvested after selfing, and the T_1_ generation was planted. Plants that tested positive for the *trans-GS5* gene according to the PCR results were self-pollinated. Each generation was planted with a single plant per hole, and the T_2_-generation seeds were obtained for PCR identification.

### 2.2. Testing of Transgenic Line(s)

The T_3_ generation of the *trans-GS5* plants were grown in a field, and 100 plants of each line were grown. A small amount of maize leaf genomic DNA was extracted for PCR amplification using the cetyltrimethylammonium bromide (CTAB) method, and a PCR reaction system (15 µL) was prepared consisting of: 30 ng of template DNA, 0.5 µL of primer GS5R (5 µmol/L), 0.5 µL of primer GS5F (5 µmol/L), 7.5 µL of 2× Taq plus Master Mix (Vazyme Biotech, Nanjing, China), and 3.5 µL of ddH_2_O. The PCR reaction program was conducted as follows: 94 °C for 5 min, 30 cycles of 94 °C for 30 s, 57 °C for 30 s, 72 °C for 1 min, and finally, 72 °C for 5 min. Electrophoresis using 1% agarose was performed to separate the PCR products, and a gel imaging system was used to detect and identify positive plants. The proportion of transgenic-positive plants in the offspring was calculated.

A specific probe, GS5R2/GS5F2, was designed to amplify a 749-bp fragment to recover the target fragment using a plasmid as a template, and the probe was digoxigenin (DIG)-labeled using a Roche kit. For lines that tested positive for the *trans-GS5* gene using PCR, high-purity bulk genomic DNA was extracted using the CTAB method. The PCR system comprised 30 µg of DNA, 4 μL of 10× L buffer, and 50 U of *KpnI*, and was supplemented with ddH_2_O to a total volume of 40 μL. The PCR system was then enzymatically digested at 37 °C for 16 h. The digested products were electrophoresed in a 0.8% agarose gel at 30 V for 16 h. The electrophoresed products were then blotted onto nylon membranes via high-salinity transfer. The hybridization and development processes were performed as per the DIG kit instructions.

RNA was extracted from the post-pollination seeds of the *trans-GS5* gene maize and the wild-type control, and reverse transcription was performed. The maize *Actin* gene was used as an internal reference for *GS5* gene expression. The RT-PCR reaction system consisted of: 2 µL of cDNA template, 0.5 µL of GS5F3 (5 µmol/L), 0.5 µL of GS5R3 (5 µmol/L), and 10 µL of 2× Taq plus Master Mix, added with ddH_2_O to a 20-µL system. The RT-PCR reaction program was conducted as follows: 94 °C for 5 min, 30 cycles of 94 °C for 30 s, 59 °C for 30 s, 72 °C for 30 s, and finally, 72 °C for 5 min. Electrophoresis using 1% agarose was performed to separate the PCR products, and a gel imaging system was used to visualize the expression.

### 2.3. Phenotypic Examination of Transgenic Line(s)

The T3 and T4 generations of the *trans-GS5* lines obtained from screening were planted in the transgenic maize experimental field of Huazhong Agricultural University for the examination of agronomic traits twice in 2 years. The T3 generation of the OE-34 and A188 lines were used as negative controls, and the F1 generation of the eight hybrid combinations with four inbred lines (Ye478, Zheng58, Chang7-2, and Huangzao4) were planted in the transgenic maize field of Huazhong Agricultural University for the observation of agronomic traits. A randomized block design was adopted for the arrangement of the experimental field, with three replications for each material, a row length of 3.0 m, a plant spacing of 0.3 m, and a row spacing of 0.6 m. Each row included 10 holes with three seeds per hole. When the materials grew to the third-leaf stage, seedlings were checked for emergence and thinned to leave one well-grown plant per hole. Plant height (height from the ground to the top of the male inflorescence) and ear height (height from the ground to the topmost female inflorescence) were measured at the mature milk stage. When the ears were mature, the plants were harvested by hand and stored by ear. The ear length (length from the base to the top of the ear), ear width (length of the middle of the ear), kernel row number (number of rows of kernels in the middle of the ear), and kernel number (number of kernels in the whole ear) were measured. After being dried, the ears were shelled and weighed. The kernel length (10 kernels in the middle of the ear were randomly selected, and length along the central axis was measured), kernel width (10 kernels in the middle of the ear were randomly selected, and length along the central axis was measured), and 100-kernel weight (100 kernels of the ear were randomly selected and weighed, with three repetitions) were also measured.

### 2.4. Determination of Starch Content in Maize Kernels

Five mature and dried kernels of the T3 generation of each line of the *trans-GS5* gene and wild-type control A188 were dried in an oven at 40 °C to a constant weight, and the weight of the seeds was recorded. The seeds were placed in a boiling water bath for 1 min, and the seed coat and embryo were carefully peeled off. The peeled embryo and endosperm were dried at 40 °C to a constant weight, and the weight of the endosperm and embryo were recorded. The total starch content of the kernels was determined using the acid hydrolysis method with a starch content assay kit.

### 2.5. Scanning Electron Microscopy of Starch Granule Morphology in Kernels

Mature kernels of the T3 generation of the *trans-GS5* gene lines OE-34 and OE-57, as well as the wild-type control material, were selected. The kernels were carefully fixed, and the tops of the kernels were tapped with a surgical scalpel to break them naturally while keeping the starch granules intact. The samples were fixed on a carrier table with double-sided tape, coated with gold for 5 min, and then, observed under a scanning electron microscope (provided by the research platform of Huazhong Agricultural University). Photographs at three different locations in the floury endosperm region of the samples were taken. The diameters of the starch granules in the floury endosperm of the maize was measured using ImageJ, and 100 starch granules were randomly selected from each photograph.

### 2.6. Starch-Synthesis-Related Gene Expression Assay

The kernels of the T3 generation of the *trans-GS5* gene lines OE-34 and OE-57, as well as the control material, 14 days after pollination were used as tissue organs for RNA extraction, purification, and reverse transcription. The maize *Actin* gene was used as an internal reference for target gene expression to examine the expression levels of a series of starch-synthesis-related genes. The RT-PCR reaction system consisted of: 2 µL of cDNA template, 0.5 µL of Primer R (5 µmol/L), 0.5 µL of Primer F (5 µmol/L), and 10 µL of 2× Taq plus Master Mix, added with ddH_2_O to a 20-µL system. The RT-PCR reaction program was conducted as follows: 94 °C for 5 min, 32 cycles of 94 °C for 30 s, 60 °C for 30 s, 72 °C for 45 s, and finally, 72 °C for 5 min. The PCR products were separated via electrophoresis using 1% agarose, and the expression was detected using a gel imaging system.

### 2.7. Determination of Kernel Weight of Maize

The T4 generation of the *trans-GS5* gene line OE-34 and the wild-type control material A188 were labeled after pollination, and the fresh weight of the 100 kernels in the middle, at different periods of 6DAP, 12DAP, 18DAP, 23DAP, and 30DAP, were weighed. The kernels were then dried in an oven at 80 °C to a constant weight, and the dry weight was measured to plot the difference curves for dry weight and fresh weight at different periods to compare the differences in grouting rates.

### 2.8. Statistics and Analysis

Microsoft Excel 2018 was used for the preliminary collation of the trial data. A one-way ANOVA and Duncan’s multiple range test were performed using SPSS software (version 22.0.0).

## 3. Results

### 3.1. Preparation of Transgenic Line(s)

**Figure 1:**
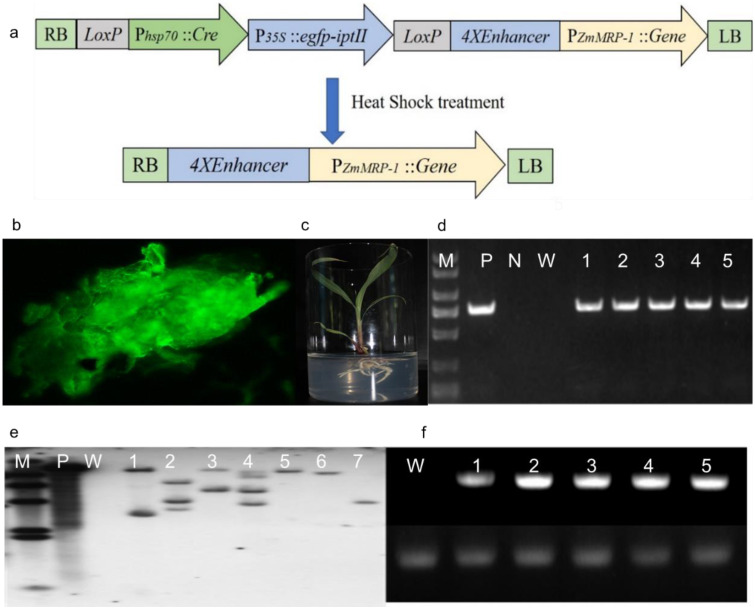
Preparation and detection of the *trans-GS5* gene in maize. (**a**) Construction of transgenic vector pHZM1N-PZmMRP-1::GS5. The pHZM1N-Rsc vector was used as the backbone [44] to construct the transgenic vector. The rice *GS5* vector expression cassette driven by the maize endosperm-specific promoter ZmMRP1 was used to replace the *Rsc* gene expression cassette driven by the ubi promoter in pHZM1N-Rsc to obtain the transgenic vector pHZM1N-PZmMRP-1::GS5. The vector was screened using the ipt-EGFP screening marker, and transgenic plants without the screening marker gene were obtained via differentiation after post-transformation heat-shock to eliminate the screening marker gene. (**b**) Screening of transgenic-positive calluses. Transgenic-positive calluses were screened using green fluorescent protein. (**c**) Obtaining marker-free transgenic plants. After heat shock to eliminate the screening markers, the transgenic calluses were differentiated to obtain normal transgenic plants, which were then transplanted to the greenhouse. *trans-GS5-*positive plants in the T_0_–T_2_ generations according to the PCR results were then selected for harvest after selfing. (**d**) PCR assay of the *GS5* gene of the T_3_ generation of transgenic maize. M: DL2000 marker; molecular-weight standard, from top to bottom: 2 kb, 1.5 kb, 1.0 kb, 750 bp, 500 bp, 200 bp, and 100 bp; P: positive control; N: negative control; W: blank control; 1–5: transgenic samples. (**e**) Southern blotting assay of the *GS5* gene in the T_3_ generation of transgenic plants. M: DNA HidIII marker; molecular-weight standard, from top to bottom: 23,130 bp, 9416 bp, 43,616,557 bp, 2322 bp, 2027 bp, and 564 bp; P: positive control; W: blank control; 1–7: transgenic samples. (**f**) RT-PCR results of the *GS5* gene in the T_3_ generation of transgenic plants. W: blank control; 1–5: transgenic samples. The upper band is the *GS5* detection band, and the lower band is the *Actin* band.

In this study, pHZM1N-PZmMRP-1::GS5, a *GS5* gene expression vector driven by the *ZmMRP1* promoter, was constructed (Figure 1a), and transgenic plants were obtained through the *Agrobacterium*-mediated transfer of maize callus (Figure 1b,c). The T_2_-generation plants were obtained after screening the two generations of T_0_ and T_1_. PCR amplification using *GS5* gene-specific primers was conducted to identify *trans*-*GS5*-positive plants among the T_3_-generation plants obtained (Figure 1d). The *trans-GS5*-positive rates for each line were 50.00% (OE-3), 44.93% (OE-6), 46.67% (OE-8), 41.18% (OE-13), 46.15% (OE-16), 78.00% (OE-), 78.00% (OE-25), 45.45% (OE-27), 22.50% (OE-30), 91.36% (OE-34), 68.29% (OE-40), 89.83% (OE-57), 44.12% (OE-62), and 68.33% (OE-70), respectively. The OE-6, OE-8, OE-13, OE-16, OE-25, OE-34, and OE-57 lines, which had relatively high positive rates, were selected for the Southern blotting assay. The results showed that all the transgenic lines had specific bands, with OE-6 and OE-13 showing two specific bands, OE-8 and OE-16 showing three specific bands, and the OE-25, OE-34, and OE-57 lines showing only one specific band (Figure 1e); this indicated that three copies were inserted in OE-8 and OE-16, two copies were inserted in OE-6 and OE-13, and a single copy was inserted into the maize genome in OE-25, OE-34, and OE-57. From these, lines with low copy-number insertions, namely OE-6, OE-13, OE-25, OE-34, and OE-57, were selected for RT-PCR analysis of the *GS5* gene. Owing to the vector design, the *GS5* gene was initiated by ZmMRP-1, an endosperm-specific promoter, and the kernels, 7 days after self-pollination, were chosen as the experimental material. The results showed that the *GS5* gene was normally expressed among the different lines, and its expression was significantly higher than that of the internal reference gene, whereas no gene expression was observed in the untransformed control (Figure 1f).

### 3.2. Examination of Agronomic Traits of Transgenic Lines

**Figure 2:**
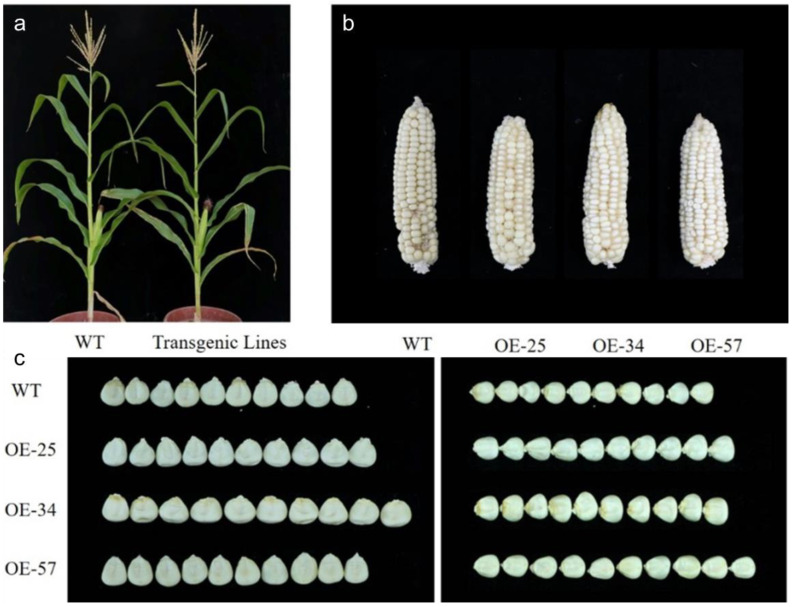
Phenotypic analysis of *GS5* transgenic maize. (**a**) Comparison of plant height at maturity of transgenic plants. (**b**) Comparison of ears of transgenic plants. (**c**) Comparison of kernel morphology of transgenic plants.

Overexpression of the *GS5* gene in both rice and wheat may increase grain width and thereby increase the thousand-grain weight. To observe the effect of the *trans-GS5* gene on maize yield, the agronomic traits of the T_3_-generation plants of *trans-GS5* gene maize were investigated (Figure 2). The studied traits were mainly yield-related traits, such as ear length, kernel row number, kernel length, kernel width, and 100-kernel weight. The investigation results are shown in Table 1. Yield-related traits were significantly improved in several lines. For example, kernel width was significantly increased in OE-25, OE-34, and OE-57, and increased from 0.73 cm to 0.78–0.80 cm, with an increase rate of 6.85–9.59%. OE-34 showed the most significant change in 100-kernel weight, with an increase of 14.10%, whereas both OE-34 and OE-57 showed highly significant increases in ear weight, with increases of 13.95% and 19.57%, respectively. However, no significant differences were observed in plant height, ear height, kernel length, kernel row number, or number of kernels. Among the different lines, no significant changes in kernel width, 100-kernel weight, or ear weight, compared to the wild type, were observed in OE-6 and OE-13, which were thus excluded from phenotypic examination in subsequent generations. Yield-related traits were also investigated in the T_4_ generation of OE-25, OE-34, and OE-57, and significant increases were observed in kernel width, 100-kernel weight, and ear weight (Table 1). By investigating yield-related traits in two consecutive generations, it was confirmed that the introduction of the exogenous *GS5* gene into maize significantly increased the kernel width, 100-kernel weight, and ear weight without significant changes to the other yield traits.

### 3.3. Comparison of Kernel Growth Dynamics and Grouting Rates of Transgenic Lines

**Figure 3:**
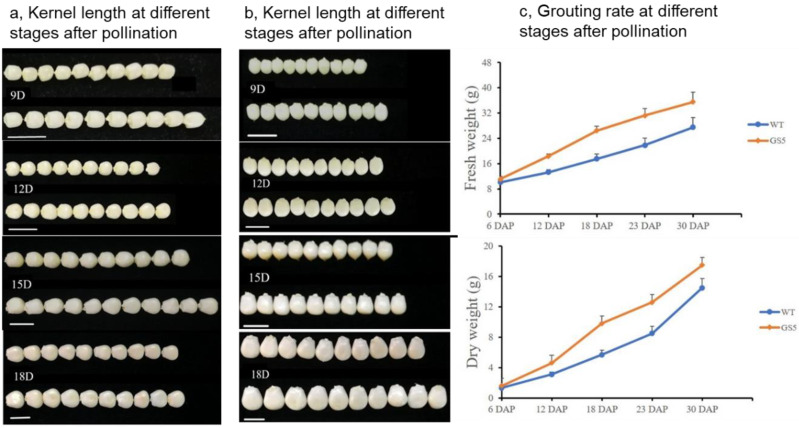
Comparison of kernel development and grouting rate dynamics between OE-34 and WT. (**a**) Dynamics of kernel length at different periods after pollination. Wild type (WT), top; OE-34, bottom; scale bar, 1 cm. (**b**) Dynamics of kernel width at different periods after pollination. WT, top; OE-25, bottom; scale bar, 1 cm. (**c**) Changes in fresh weight and dry weight of kernels on different days after pollination.

The test results showed that several yield traits, such as 100-kernel weight, kernel width, and yield per plant, were significantly higher in the *GS5* transgenic line OE-34 than in the wild type. To clarify the effect of the *GS5* gene on maize kernel development, OE-34 was selected for further experimentation, with the wild-type A188 line used as the control material, to compare the developmental dynamics of kernel length and kernel width after pollination. There were significant differences in kernel width 9–18 days after pollination (Figure 3a), but significant differences in kernel length were only observed on the 9th and 15th day after pollination. On the 18th day after pollination, there was a significant difference in kernel width, but no significant difference in kernel length was observed between the two (Figure 3b). The kernels in the middle of the ear of the transgenic line OE-34 and the wild-type control A188 line at different periods after pollination were obtained to count the fresh and dry weights, to compare the differences in grouting rate based on 100-kernel weight. The results showed that OE-34 had higher dry weight and fresh weight than the wild type 6–30 days after pollination, and the difference was more significant in the later stages of kernel development; this indicates that the overexpression of the *GS5* gene in maize increased the grouting rate, and that the difference in grouting rate was more obvious during the later stages of grain development (Figure 3c).

### 3.4. Effect of the Trans-Gs5 Gene on Starch Synthesis in Maize Kernels

Starch is the most important storage material in maize kernels, accounting for approximately 70–80% of the kernel weight. The endosperm is the site of starch synthesis and storage in maize, and is closely related to the weight of maize kernels. To verify whether the *GS5* gene affects starch content in the endosperm of the kernels, the acid hydrolysis method was adopted for starch content determination. Our results showed that *GS5* gene overexpression significantly increased the starch content of the kernels, with the starch content of OE-25, OE-34, and OE-57 increased by 10.30%, 7.39%, and 6.39%, respectively (Table 2).

**Figure 4:**
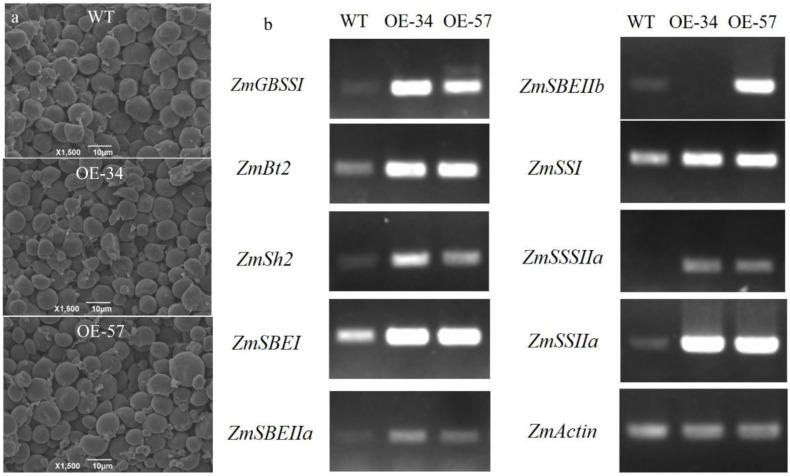
Morphology of starch granules (**a**) and expression of genes related to the starch synthesis pathway in mature kernels of T_3_-generation *trans-GS5* maize (**b**).

Table 2 shows that in the materials with *GS5* gene overexpression, the starch content was elevated by up to approximately 10%. Starch in the maize kernels existed as starch granules. To clearly visualize whether the starch granules changed in size, scanning electron microscopy was performed to observe the starch granule morphology in the floury endosperm region of mature kernels in the both transgenic and control materials. Scanning electron microscopy images showed that the starch granules of the two were either round or oval, whereas the starch granules of the material with *GS5* gene overexpression were highly heterogeneous in size (Figure 4a). Measurement of the starch granule diameters revealed that most starch granules in the transgenic line(s) were in group a (starch granule diameter < 9 μm) compared with those in the wild-type material (wild type, 56.36%; OE-34, 87.25%; OE-57, 84.67%), and significantly fewer starch granules were in group b (9–13 μm) (wild type, 43.64%; OE-34 12.75%; OE-57, 14.67%). The average diameter of the starch granules was reduced. This suggests that the *GS5* gene may have elevated starch content in the trans-*GS5* lines by affecting the size of the starch granules.

Four key classes of enzymes, namely ADP-glucose pyrophosphorylase (AGPase), starch synthase (SS), starch branching enzyme (SBE), and debranching enzyme (DBE), together regulate starch synthesis in maize. As the introduction of the exogenous *GS5* gene in maize affected starch content and starch granule morphology, kernels were selected 14 days after pollination as experimental materials for semiquantitative RT-PCR to detect the expression of key starch synthesis genes in maize and analyze the effect of the *GS5* gene on the starch synthesis pathway in maize kernels. The results showed that the expression of *ZmGBSSI*, *ZmSh2*, *ZmBt2*, *ZmSBEI*, *ZmSBEIIa*, *ZmSBEIIb*, *ZmSBE*, *ZmSSI*, *ZmSSIIa*, and *ZmSSIIIa* in the transgenic line(s) were significantly different from that in the non-transgenic control, and that the overall expression of these starch-related genes increased; this indicates that the *GS5* gene may have acted as a positive regulator in transgenic maize by promoting the expression of related genes in the starch synthesis pathway, which led to changes in the diameter and size of the starch granules, thus increasing the starch content.

### 3.5. Effects of the Trans-Gs5 Gene on Maize Breeding

**Figure 5:**
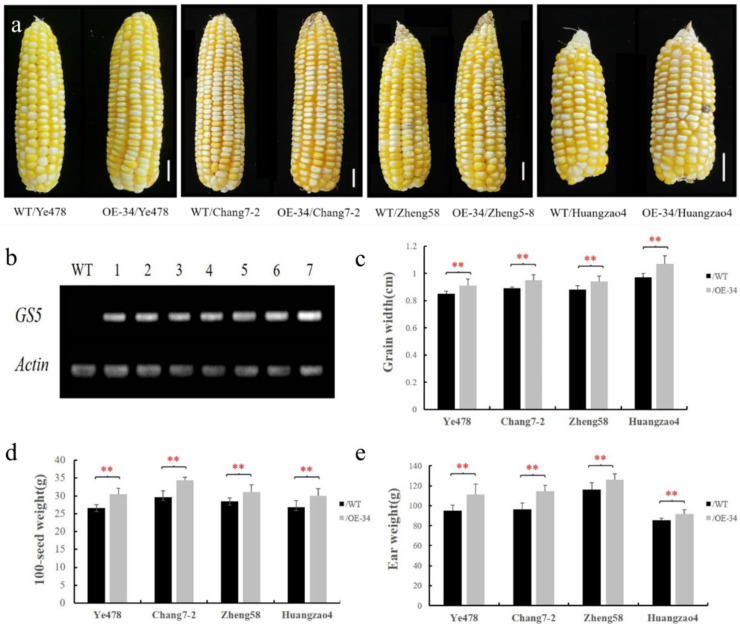
Evaluation of yield-related traits in GS5-OE-34 hybrids. (**a**) Phenotypic plots of ears of F_1_-generation GS5-OE-34 hybrids. Scale bar, 2 cm. (**b**) *GS5* expression levels in the F_1_ generation of GS5-OE-34 and its corresponding wild type crossed with Ye478, Zheng58, Chang7-2, and Huangzao4. (**c**–**e**) Phenotypic analysis of kernel width, 100-kernel weight, and ear thickness in hybrids. Bar indicates means, and error lines represent standard deviations. ** represents a significant difference at *p* < 0.01.

The inbred line A188 has been widely used as a recipient for maize transformation owing to the easy induction of its callus and high transformation efficiency. However, because of its shortness and low yield, A188 cannot be used as a parent for breeding high-yielding maize. Therefore, the *GS5* transgenic line OE-34 and the non-transgenic inbred line A188 (negative control) were used as the male parents, and elite inbred lines commonly used in breeding, namely Zheng58, Chang7-2, Ye478, and Huangzao4, were used as the female parents for crossing. The F_1_-generation crosses produced were used to examine yield-related traits, and to assess the effect of the *GS5* gene on maize breeding. The RT-PCR results showed that the *GS5* gene was highly expressed in all crosses, which indicated that *GS5* gene expression was stabilized in transgenic maize (Figure 5b). Phenotypic characterization was conducted for the eight crosses, and the results showed that the crosses containing *GS5* had significantly increased kernel width, 100-kernel weight, and ear weight compared with the respective non-transgenic controls (Figure 5c–e), with a 5.85–10.02% increase in kernel width, a 9.07–15.90% increase in 100-kernel weight, and a 7.77–17.01% increase in ear weight. However, no significant differences were observed in plant height, ear height, ear length, kernel length, kernel row number, or number of kernels. These results indicate that the *GS5* gene can indeed change the kernel shape and increase the kernel weight in maize. Moreover, the results proved that the next step of backcrossing into the elite inbred lines should be effective.

## 4. Discussion

Genetic improvement to obtain more and better varieties of crops, such as maize, suitable for various purposes is a constant theme in agricultural production [45,46]. Although significant achievements have been made in traditional crop breeding, one shortcoming has gradually emerged, namely the inability to break the interspecies barrier [47,48,49]. When genes encoding a certain quality trait are not available within a crop, it is difficult to improve such a trait [28,50,51]. Transgenic technology has developed with the advancement of modern biotechnology and has broken reproductive isolation between species, enabling the introduction of various exogenous genes to crops [52,53,54,55]. The expression and function of exogenous genes in the new recipient plants have improved diverse genetic traits in crops [56,57,58]. In this study, the rice *GS5* gene was introduced to maize using transgenic technology, and monocopy lines with high *GS5* expression were selected. The phenotypic identification of two generations indicated that kernel width and 100-kernel weight were significantly improved in the *GS5* transgenic lines. Yield-related traits were examined in the F_1_ generation of *GS5* transgenic lines with different material backgrounds, and it was found that the *GS5*-overexpressing hybrids had significantly increased kernel width and 100-kernel weight. In contrast, there were no significant changes in the other agronomic traits of transgenic maize, which laid a solid foundation for breeding new high-yielding transgenic maize varieties with increased kernel weight.

Starch is the main component of maize kernels [59,60]. After the introduction of the exogenous *GS5* gene to maize, the starch content of maize kernels increased from 66.30% to 72.59–76.98%, with significant changes in the morphology of starch granules. In addition, RT-PCR showed changes in the expression of genes related to starch synthesis. The *GS5* gene is a major gene regulating the grain width, thousand-grain weight, and grouting rate of rice seeds, and it was isolated and identified in rice using map-based cloning and other methods. The action mechanism of the *GS5* gene in rice has not been well understood, and only preliminary evidence is available [61,62]. The rice *GS5* gene encodes SCPLs. Functional studies of this class of protein in barley, wheat, *Arabidopsis thaliana*, and rice have shown that SCPLs play a role in many processes such as the hydrolysis of storage proteins during seed germination, the autolysis of cellular components in programmed cell death, seed development, and stress resistance [63,64]. In rice, elevated *GS5* expression leads to the accumulation of a large amount of *GS5* protein in the extracellular structural domain of *OsBAK1*; this prevents *OsMSBP1* [65] from interacting with *OsBAK1* and maintains *OsBAK1* on the cell membrane, thereby facilitating OsBARI1–OsBAK1 interactions [66,67,68]. The OsBARI1-OsBAK1 interactions enhance brassinosteroid signaling and subsequently promote cell division in the inner and outer glumes, resulting in phenotypes with wider seeds and increased thousand-grain weight [69,70]. However, some of the results remain to be validated. We speculated that the overexpression of the *GS5* gene in maize may have also promoted brassinosteroid signaling in maize, which enhanced the source of the “sink” and increased the accumulation of photosynthetic products by regulating the source–sink balance of photosynthetic products [71,72,73]. In addition, the endosperm-specific promoter used in this study was able to strongly promote the uptake of photosynthetic products into the endosperm transfer cell layer, which increased the uptake of soluble sugars and accelerated the synthesis rate of the starch substrate, ultimately leading to a phenotype with increased starch content, wider kernels, and higher kernel weights [74,75,76]. These assumptions may be validated by subsequent in-depth studies. Aside from providing the basic materials for the breeding of high-yielding maize, the transgenic maize harboring the rice *GS5* gene may also provide scientific research materials and clues to elucidating the action mechanism of the *GS5* gene.

Gene promoters are one of the important factors affecting gene transcription levels, and thus, they determine the traits regulated by the related genes [77,78,79]. The use of promoters and enhancers with specific regulatory effects is essential for the efficiency of exogenous gene expression in recipient plants. The constitutive expression promoters such as rice *Actin1* [80,81], maize *Ubi* [82,83], and 35S [84,85,86] has been used in plant transgenic engineering. However, these promoters tended to be transcribed in all plant tissues with poor spatio-temporal specificity, which increased plant energy consumption and also tended to result in gene silencing [87,88]. Multiple studies have shown that the selection of a suitable promoter specific to a particular tissue may avoid unintended phenotypes and reduce plant energy consumption to ensure accurate and high expression of the target gene at the desired location. In this study, the target genes *GS5* and *ZmMRP-1* were driven by promoters derived from maize itself, with four 35S enhancer sequences in tandem ahead of the promoters [89,90]. The enhancer 35S only enhanced the expression of neighboring genes, without altering the spatio-temporal specificity of adjacent genes, to ensure that the *GS5* and *ZmMRP-1* genes could be expressed efficiently at the critical period and critical site of grain weight formation. A gene expression assay of the positive *ZmMRP-1* transgenic maize at different periods suggested that *ZmMRP-1* was overexpressed only in the endosperm after pollination and reached its peak on the 12^th^ day after pollination. In addition, the agronomic traits of the *ZmMRP-1*-positive maize were not significantly different from those of the negative control, which indirectly indicated that the trans-*ZmMRP-1* gene did not interfere with the development of other parts of the maize.

## 5. Conclusions

Maize is one of the most important food crops, and corn grain is an important part of the yield. As the main storage material of endosperm, endosperm starch content is the key to determining grain yield. In this study, the rice *GS5* gene, induced by an endosperm-specific promoter was transferred into maize through agrobacterium-mediated genetic transformation. Through a series of experiments such as molecular detection, expression analysis, corn kernel development detection, and yield determination of the target gene, it is proven that the gene is expressed in corn endosperm; this can improve corn kernel yield by regulating starch synthesis, and finally, improve the yield of corn combinations. This study provides an example of further using genetic transformation to improve maize, and also provides excellent materials for maize yield improvement.

## Figures and Tables

**Table 1:** Agronomic traits of T_3_-generation *GS5* transgenic maize.

Lines	Plant height	Ear height	Ear length	Ear diameter	Ear rows
WT	137.97 ± 9.49	33.57 ± 5.45	12.31 ± 1.58	3.61 ± 0.23	13.43 ± 1.51
OE-6	135.04 ± 9.60	35.11 ± 4.72	11.99 ± 1.09	3.54 ± 0.26	13.14 ± 1.07
OE-13	131.81 ± 12.04	34.03 ± 7.60	12.03 ± 1.02	3.53 ± 0.51	13.14 ± 1.07
OE-25	132.59 ± 11.13	33.35 ± 4.25	12.60 ± 0.80	4.30 ± 0.57 **	13.67 ± 1.89
OE-34	132.63 ± 9.40	33.85 ± 3.79	12.30 ± 0.58	4.17 ± 0.49 *	12.86 ± 1.07
OE-57	136.69 ± 16.69	34.18 ± 9.55	13.21 ± 1.64	3.82 ± 0.65	12.57 ± 0.98
Lines	Kernel number per ear	Grain length	Grain width	100-seed weight	Ear weight
WT	270.71 ± 43.69	0.86 ± 0.06	0.73 ± 0.08	17.30 ± 2.36	59.47 ± 9.86
OE-6	261.14 ± 32.17	0.87 ± 0.04	0.72 ± 0.02	16.83 ± 1.91	58.50 ± 7.39
OE-13	266.86 ± 17.46	0.86 ± 0.04	0.75 ± 0.03	17.28 ± 0.51	54.36 ± 5.23
OE-25	299.83 ± 30.07	0.89 ± 0.15	0.79 ± 0.03 **	17.78 ± 1.33	65.05 ± 8.87
OE-34	265.71 ± 28.50	0.91 ± 0.04	0.80 ± 0.05 **	19.74 ± 0.78 **	67.77 ± 5.07 **
OE-57	294.86 ± 68.24	0.90 ± 0.07	0.78 ± 0.10 *	18.81 ± 0.87	71.11 ± 9.85 **

Values are expressed as means ± standard deviations, n = 18. * represents a significant difference at *p* < 0.05, whereas ** represents a significant difference at *p* < 0.01.

**Table 2:** Comparison of starch content and endosperm size of kernels of T_3_-generation *GS5* transgenic maize.

Lines	Starch Content (%)	Endosperm Weight (mg)	Embryo Weight (mg)	Endosperm/Embryo
WT	66.30 ± 0.01 a	142.20 ± 2.34 a	31.61 ± 1.22 a	4.50 ± 0.10 a
OE-25	76.99 ± 0.01 c	189.90 ± 2.34 b	35.50 ± 2.98 b	5.35 ± 0.14 b
OE-34	73.59 ± 0.01 b	196.53 ± 2.26 b	36.90 ± 1.70 b	5.33 ± 0.21 b
OE-57	72.59 ± 0.01 b	186.75 ± 1.26 b	36.70 ± 3.02 b	5.09 ± 0.17 b

Values are expressed as means ± standard deviations. Different letters represent significant differences (*p* < 0.05, Duncan’s test, n = 5).

## Data Availability

Not applicable.
